# Selection of Characteristic Symptoms

**Published:** 1888-08

**Authors:** Edward Cranch


### SELECTION OF CHARACTERISTIC SYMPTOMS; HAHNEMANN'S "ORGANON," PARAGRAPH 153.

Dr. Lippe, whose transference to a different sphere of usefulness we all mourn, missing him as a departed teacher, guide, and friend, assigned the special subject of the present paper while making up this bureau last summer.

A careful translation of paragraph 153 with the sentence just before it reads as follows: "Among the symptom-lists of many medicines it should not be difficult to find *one* out of whose separate pathogenetic elements it is possible to arrange a picture of a curative, artificial disease, very like to the sum-total of the symptoms of the natural disease, and this medicine is the most desirable remedy. In this search for homœopathic specific remedy, that is, in this comparison of the total signs of the natural sickness with the lists of symptoms of available drugs in order to find among these *one* bearing a pathogenetic power corresponding to and resembling the disease to be cured, the *striking*, *remarkable*, *uncommon*, and *peculiar* (characteristic) signs and symptoms of the case of sickness are to be especially and almost exclusively brought before the eye, for *these especially must be very like the drug that is being searched for in the symptom-lists* if this is to be the most suitable for the cure. The general and indefinite, such as loss of appetite, headache, weakness, restless sleep, discomfort, etc., if they are not more closely defined, deserve little attention, for one finds something about as indefinite in almost every sickness, and caused by almost every drug."

As Dr. Wells showed years ago in a paper read before the Central New York Homœopathic Society, this section is a corollary to paragraph 18, which reads as follows : " From this not-to-be-doubted truth that, outside of the assemblage of symptoms, there is no way to find out anything about diseases by which their need of assistance can be declared, it follows, undeniably, *that the totality of all positively known symptoms* in every single case of sickness must be the *sole indication*, the only hint, in the choice of a remedy."

Here is an apparent but not a real contradiction, for the latter paragraph only shows us how to *use* the totality of symptoms. If we would successfully select the characteristic symptoms of the case and of the medicine we must not shut our eyes to that which is general and universal. We must *see* the *totality* and *use* it with the *peculiarity*.

Dr. Lee having prepared a paper for this meeting on the characteristics as understood by Hahnemann, this paper will only give some thoughts on the ways of looking for and recording characteristics with a view to the gradual improvement of the working value of the *Materia Medica*.

First, we must, as Dr. Lippe often asserted, understand pathology that we may know how to examine the patient intelligently, but we must be careful not to let our minds rest in merely pathological expressions or we shall lose much that is of the highest value, besides wasting our time in referring symptoms to pathological states, and perhaps, after all, do this erroneously.

Dr. T. F. Allen, in defending his retention of the Hahnemannian arrangement of symptoms, said it would outlive all theories, for it was a plain record of observations. In taking notes of a case of sickness or of a proving, we want only the exact record of phenomena observed. What matters it how many cases of pneumonia or of rheumatism we have seen get well? Would that information help us to treat the next case of either? But if the condition of mental anguish, thirst, fever, and restlessness, as caused by Aconite, has always been cured by Aconite when existing as a similar condition in the sick, is it not our quickest and surest plan to note and remember this coincidence and exhibit Aconite *whenever* we encounter similar conditions?

Pathology is of use to all physicians every day, but it is not of much value in the exact recording of symptoms, and nearly all conditions of the sick can be better described without referring to its terms and idioms.

To illustrate some of the real difficulties that lie in the way of a satisfactory selection of symptoms that are to be considered characteristic, the results of experience in the writer's practice for the month of March last may not be unprofitable, although they involve confessions of weakness not always easily accounted for.

In the aforesaid month of March six hundred and sixty-three prescriptions were made, of which only twenty-two escaped record, leaving six hundred and forty-one to be considered.

In three hundred and sixty-seven, or a little more than half of these, the indications found were vague, that is to say, not clearly characteristic, and therefore not wholly pleasing. The reasons for this unwelcome vagueness are to be classified under several heads:

I. Insufficient information; the symptoms being furnished by letter or messenger, or occurring in those who were not of a proper age or state of mind to bear cross-examination. Sometimes the most careful questioning and examination elicited nothing beyond the first general statement of condition. (On this head, see Sections 176 to 180, where Hahnemann directs the nearest possible remedy to be given, which he says will develop the latest symptoms of the case, ready for a new prescription.)

II. Confused state of questioner, by reason of haste, fatigue, impatience, distracting noises, preoccupation of mind, or possible laziness.

III. Insufficient knowledge of materia medica, by which possibly prominent characteristics escaped notice.

IV. Imperfect state of, or too hasty use of the repertories, by reason of which partly remembered symptoms escaped the searcher. This last condition will be greatly mitigated on the appearance of Dr. Lee's forthcoming *Repertory of Characteristics*, but the conviction is strong on the writer that every one should write a repertory of his own besides.

Of these three hundred and sixty-seven vague prescriptions, one hundred and seventy-nine helped, or seemed to help, the patients who took them; one hundred and four failed, more or less conspicuously, and the remainder, eighty-four, have not yet been heard from.

There were one hundred and one prescriptions of placebo, on account of visible improvement from previous prescriptions.

The remaining one hundred and seventy-three, less than one-fourth of the whole number, presented clear characteristics, satisfactory at the time to the writer; of these, one hundred and forty-seven, or nearly all, succeeded in bringing relief, generally with great promptness; only ten failed, as far as heard from, and sixteen remained unreported.

Leaving out the cases receiving only placebo, we have a total of five hundred and forty (representing one hundred and ninety-eight separate cases of sickness) in which the writer's average efforts were exerted to find the appropriate homœopathic remedies. That only one hundred and seventy-three were clear, and that ten of these distinctly failed, is not very encouraging, except as an incentive to better work in the future. Of the one hundred and forty-seven cures by means of characteristics that were encountered, only ten presented striking symptoms that were apparently new to the drugs used. These new symptoms cured were the following:

*Actea racemosa*$^{1000}$.—"The recently delivered uterus becomes actually jammed into pelvis, with great pain."

*Arsenicum*$^{1000}$.—"Daily nosebleed, mornings."

*Belladonna*$^{1000}$.—"Headache worse, and hurts if leaned against anything."

*Carbo animalis*$^{30}$.—"Parotid gland swells every day at sundown, better all day."

*Carbo animalis*$^{30}$.—"Throat felt as if she had swallowed a piece of paper."

*Carbo animalis*$^{30}$.—"Region of throat behind palate aches severely without other soreness."

*Lithium carbonicum*$^{200}$.—"Sour stomach from tomato soup."

*Ranunculus bulbosus*$^{200}$.—"Pain like that of shingles, without eruption."

*Millefolium*$^{30}$.—"Menorrhagia while baby nurses if beer is drank."

*Pulsatilla*.—"Nervousness felt, especially about ankles."

In all these cases, these *odd* symptoms were very striking in the patient, and corroborated by other well-known indications. The rest of the symptoms were only verifications of old symptoms, not to be slighted on that account, but only for lack of space for the whole number.

The ten failures were upon the following indications:

*Bryonia*.—Nausea on rising.

*Calcarea phos*.—Has to strain long for even a soft stool.

*Carbo animalis*.—Effects of strain of back.

*Hyoscyamus*.—Cough only and always on lying down.

*Kali carbonicum*.—Needle-like pains all over left arm.

*Stramonium*.—Sees horrid visions, fears the dark.

*Sulphur*.—Heat of vertex, with latent eczema.

These cases show the fallacy of relying too much on single symptoms, however suggestive. Hahnemann was very careful to tell us to look for the *striking*, *uncommon*, and *peculiar symptoms*, not the *one most peculiar symptom*. And these, he says, *must be very like the drug*. It follows, then, that any set of symptoms to be of the highest value, must bear equal rank in the patient and in the proving; that is, however peculiar a symptom is to a drug, as far as known, if it is not equally striking and peculiar in the case in hand, and supported by a general likeness to the constitutional action of the medicine, it will fail. Dr. Ballard, our Secretary, well illustrated this point in an article in the *Medical Current*, for April 20th, 1887, where he showed that Cina failed on one of its peculiarities, "sleep only during violent rocking." Calcarea cured, because it suited better the general diathesis which was peculiar to the case, and really the most strikingly prominent sign of the child's disease. So it will always be that we must assemble all available symptoms of the case, and from them all endeavor to select the *most* peculiar, not forgetting the others, but seeking corroboration from them, especially when the most striking symptoms point to several remedies at once. In such cases, as Dr. Dunham has shown, some one remedy can generally be found whose picture is the nearest counterpart to the picture of the disease, although it may differ in a few features. Thus copious eructations of gas suggest *Carbo veg.*, great weakness suggests also *Arsenicum*, general irascibility suggests *Nux vomica*, sensitiveness to cold suggests *Hepar sulph.* One case presented all these symptoms, but in addition a regular *evening foreboding*, prophesying all sorts of ills, and the air eructated was sour instead of burning. *Calcarea carb.*$^{1000}$ in one dose caused the prompt removal of all symptoms. Two months later they returned, without the evening forebodings, and *Nux vomica*$^{30}$ relieved with equal promptness.

If we could find a list of indispensable symptoms, without one or more of which certain drugs would be absolutely contra-indicated, as suggested concerning the mental symptoms of Aconite, Nux vomica, Pulsatilla, and Ignatia in Hahnemann's note to Section 213, our task would be much simplified, but until our provings have greatly increased in number and fullness, such a list is likely to remain imperfect. Therefore, let us endeavor to study and repeat provings, till every drug we can find is accurately pictured. The examination of the patient is still more difficult, for as soon as one striking symptom has suggested a drug, the imagination too easily fills up the picture, instead of relying solely on observation, which may, after all, discover a more closely related remedy. Only the observations *that can be expressed in words* are, as a rule, reliable. The flitting fancies of the mind make sorry will-o'-the-wisps, and lead us into many hasty errors, afterward sadly regretted and hard to recall.

All striking, remarkable, uncommon, and peculiar symptoms should find a place in our notes, and those that are new and cured, if often verified, deserve a place as "Clinical Symptoms," to await further verification in some future proving. It is useless to burden the note-book with names of diseases or pathological guesses; they mislead far oftener than they help.

However undesirable it may be, the fact remains that time will not always allow the noting of every case in the careful manner directed by Hahnemann, but frequent practice in such work trains the mind to arrange the symptoms mentally in order, and the busiest practitioner can put down at least a summary; that is to say, the most striking, remarkable, uncommon, and characteristic signs and symptoms of the case. Thus only will he by and by be able, occasionally, to predict the result of a given prescription with assured confidence, and to rejoice in the result.

EDWARD CRANCH.

